# Investigating the role of melanocortinergic, glutamatergic and neuropeptide Y systems on hypophagia caused by gastric inhibitory polypeptide (GIP) in broilers

**DOI:** 10.1016/j.psj.2025.106324

**Published:** 2025-12-19

**Authors:** Maryam Lotfi Gharaie, Morteza Zendehdel, Hamed Zarei, Kimia Mahdavi

**Affiliations:** a Department of Basic Sciences, Faculty of Veterinary Medicine, University of Tehran, Tehran, Iran; b Department of Biology, CT.C., Islamic Azad University, Tehran, Iran

**Keywords:** Appetite, Gastric inhibitory polypeptide, Neuropeptide Y, Melanocortin, Glutamate

## Abstract

Appetite control and the metabolic rate of energy expenditure are under the influence of a broad suite of biochemical messengers, which include peptide-based signals, hormonal agents, and neurotransmitters. This research sought to examine the correlation between melanocortinergic, glutamatergic, and neuropeptide Y (NPY) systems with gastric inhibitory polypeptide (GIP) in the modulation of appetite and consummatory behavior. In experiment 1, broilers were injected with GIP at various doses (3, 6 and 12 nmol) in addition to saline. The experimental design for the second trial involved four distinct ICV treatment groups: a saline control, SHU9119 (a MC3/4 melanocortin receptor antagonist, 0.5 nmol), GIP (12 nmol), and a co-infusion of SHU9119 and GIP. Experiments 3 through 11 followed a protocol analogous to that of experiment 2. The sole modification was the substitution of SHU9119 with specific receptor antagonists, administered via intracerebroventricular (ICV) injection. These antagonists targeted the following receptors and their respective doses: MC4 (HS024, 0.5 nmol), NMDA (MK-801, 15 nmol), AMPA (CNQX, 390 nmol), mGluR1 (AIDA, 2 nmol), mGluR2 (LY341495, 150 nmol), mGluR3 (UBP1112, 2 nmol), NPY1 (BMS193885, 1.25 µg), NPY2 (CYM9484, 1.25 µg), and NPY5 (L-152804, 1.25 µg). Cumulative feed intake was subsequently measured at 30, 60, and 120-minute post-infusion intervals. Based on the results, GIP at doses of 6 and 12 nmol induced a significant reduction in food consumption relative to the control group (*P* < 0.05). This anorexigenic effect of GIP was significantly attenuated by the co-infusion of either the MC3/MC4 receptor antagonist SHU9119 (*P* < 0.05) or the NMDA receptor antagonist MK-801 (*P* < 0.05). The collected data imply that signaling cascades dependent on NMDA and melanocortin (MC3/MC4) receptors potentially mediate the anorectic effect of GIP in neonatal broilers, a process which appears independent of NPY Y1, Y2, and Y5 receptor involvement.

## Graphical abstract

**Figure:**
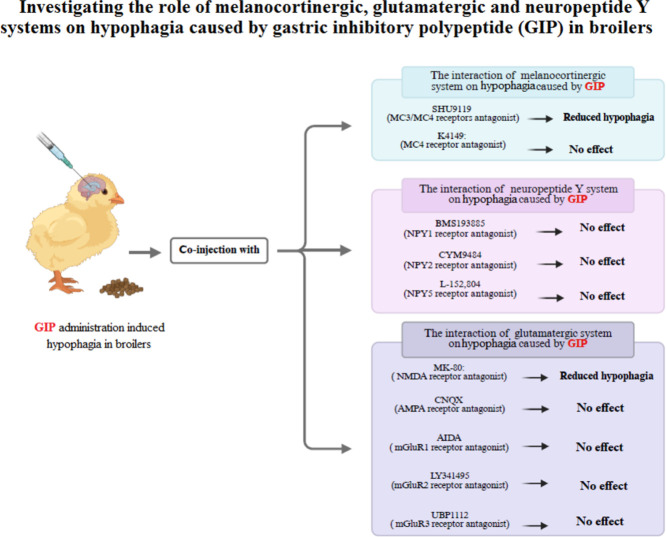

## Introduction

In broiler production systems, the control of ingestive behavior is a key factor underpinning productivity, with direct implications for growth efficiency and final body conformation (Mohammadabadi et al., 2021). Intensive genetic selection in broilers has enhanced growth but also predisposed them to hyperphagia, which can lead to excessive fat deposition and metabolic disorders (Tavárez and Solis de los Santos, 2016; Honda, 2021; Jie et al., 2024). Therefore, understanding the neurobiological mechanisms controlling feed intake is of paramount importance. This complex process is centrally coordinated by the hypothalamus, which serves as the primary central hub for energy homeostasis, integrating peripheral signals to regulate food intake (Palkovits, 2003). Within the hypothalamus, the arcuate nucleus (ARC) plays a pivotal role, housing two key populations of neurons with opposing effects on appetite: the orexigenic NPY/AgRP neurons and the anorexigenic POMC neurons (Na et al., 2022; Roh and Choi, 2023).

These hypothalamic circuits are modulated by numerous hormonal signals, including those originating from the gastrointestinal tract. Among gut-brain signals, gastric inhibitory polypeptide (GIP) is a metabolically versatile hormone. Beyond its peripheral roles (Baggio and Drucker, 2007), GIP acts centrally to regulate energy balance. GIP receptor (GIPR) expression is localized within a distributed network of central nuclei governing energy balance, including key hypothalamic sites like the ARC, paraventricular (PVN), and dorsomedial (DMN) nuclei, as well as brainstem centers (Yada, 2024). Intracerebroventricular (ICV) administration of GIP suppresses meal consumption in a dose-dependent manner in mice (NamKoong et al., 2017). Although bioinformatic analyses confirm GIP gene expression in birds (Irwin and Zhang, 2006), the precise physiological role of GIP, especially in feeding behavior, remains poorly understood in avian models, highlighting a critical gap in our knowledge.

To elucidate the central mechanism of GIP, it is essential to investigate its interaction with established hypothalamic appetite-regulating pathways. The melanocortin system, primarily via melanocortin-3 and −4 receptors (MC3R and MC4R) in the hypothalamus, is a well-established anorexigenic pathway in both mammals and birds (Strader et al., 2003; Schneeberger et al., 2014; Bungo et al., 2011). Its activity is critically modulated by key metabolic signals, including leptin and insulin (Boswell and Dunn, 2015; Ehrhardt et al., 2023), which regulate the balance between NPY/AgRP and POMC neurons in the ARC.

Beyond peptidergic systems, classic neurotransmitters also play a fundamental role in central appetite control. Glutamate, the primary excitatory neurotransmitter within the CNS, also serves as a key anorexigenic signal in the avian brain. Its receptors are categorized into ionotropic (NMDA, AMPA) and metabotropic (mGluR I, II, III) classes (Shojaei et al., 2020). Central administration of glutamate via the ICV route produces a significant, dose-dependent suppression of meal consumption in neonatal broiler chicks, while antagonism of metabotropic glutamate receptors stimulates food intake (Ahmadi et al., 2019; Baghbanzadeh and Babapour, 2007).

In stark contrast, NPY represents a highly conserved and potent stimulator of appetite. It orchestrates its powerful hunger-promoting effects primarily through G-protein-coupled receptors, particularly the Y1, Y2, and Y5 subtypes being critically implicated in stimulating meal intake (Zahed et al., 2024; Mahdavi et al., 2024). Central administration of NPY potently and reliably induces hyperphagia in mammalian models (Gumbs et al., 2022), a robust orexigenic action conserved in avian species (Greene et al., 2022). The primary site for NPY's orexigenic action is the hypothalamic ARC, where a distinct population of neurons co-expresses NPY and AgRP (Na et al., 2022). Given the established role of Y1, Y2, and Y5 receptors as the primary mediators of NPY's orexigenic signal, this study focused on investigating the potential interaction between GIP and these specific receptor subtypes.

Notwithstanding the established roles of these individual systems, the potential interaction of GIP with these central pathways remains largely unexplored in birds. Studies have revealed that GIP interacts with the melanocortin system, as GIP receptor activation modulates POMC neuronal activity in the arcuate nucleus (Yada, 2024). Furthermore, GIP demonstrates crosstalk with NPY, where central NPY administration has been shown to stimulate GIP secretion, suggesting a bidirectional communication (Yavropoulou et al., 2008). Additionally, the widespread expression of GIP receptors in brain regions rich in glutamatergic synapses implies potential interaction with this major excitatory system (Adriaenssens et al., 2019). However, the functional significance of these interactions with the melanocortin, NPY, and glutamatergic systems in the control of meal consumption, particularly in avian species, remains largely unexplored. This research aimed to synthesize the interplay between GIP and the melanocortinergic, glutamatergic, and NPY systems, thereby examining new evidence on how GIP potentially modulates food consumption in broilers. Specifically regarding NPY, this study focused on its primary orexigenic receptor subtypes (Y1, Y2, and Y5) to investigate their potential role in GIP-induced hypophagia.

## Materials and methods

### Ethical Declaration

All experimental protocols were performed in strict compliance with the National Institutes of Health's Guide for the Care and Use of Laboratory Animals (NIH publication No. 85-23, revised 1996) and applicable national legislation on animal research. The study design received formal approval from the Institutional Animal Ethics Committee of the Faculty of Veterinary Medicine, University of Tehran (Approval No. IR.UT.VET.REC.1403.061). Every reasonable effort was made to alleviate animal distress and to utilize the minimum number of birds required to obtain scientifically valid results.

### Animals and husbandry protocol

This investigation utilized 528 one-day-old male Ross-308 broiler chicks obtained from a commercial hatchery (Mahan Company, Tehran, Iran). Upon arrival, broilers were group-housed in floor pens (2.0 *m* × 1.5 m) on wood shaving litter at a density of 25 chicks per pen for a 48-hour acclimation period. The climatic conditions within the chamber were precisely controlled, sustaining a constant temperature of 32°C and a relative humidity of 40–50%.

After acclimation, birds were randomly assigned to individual wire cages (30 cm *W* × 40 cm *L* × 40 cm H). All nutritional requirements were met ad libitum via a dedicated linear feeder (10-cm) and a cup waterer (250-mL) provisioned with a commercial starter formulation (21% crude protein and 2850 kcal/kg metabolizable energy; Chineh Company, Tehran, Iran) and fresh water, respectively. To ensure accurate measurement of feed intake, a collection tray was placed under each cage; any spilled feed was carefully collected, weighed, and subtracted from the total feed provided.

Environmental conditions were strictly controlled throughout the study. A 23L:1D photoperiod was maintained with a light intensity of approximately 20 lux at bird level. Ventilation was provided by a central automated system ensuring 8-10 air changes per hour. All experimental procedures, including intracerebroventricular (ICV) injections and subsequent feed intake monitoring, were conducted within a standardized time window (08:00 to 15:30) to minimize potential confounding effects of circadian rhythms (Furuse et al., 1997).

### Experimental drugs

The following materials were acquired from Sigma Co. (Saint Louis, MO): synthetic mouse GIP (YAEGTFISDYSIAMDKIRQQDFVNWLLAQRGKKSDWKHNITQ), which was used due to the lack of a commercially available avian GIP; specific antagonists for NPY receptors (B5063 for Y1, SF22 for Y2, SML0891 for Y5); melanocortin receptor antagonists (SHU9119 for MC3/4, HS024 for MC4); glutamate receptor antagonists (CNQX for AMPA, MK-801 for NMDA, AIDA for mGluR1, LY341495 for mGluR2, UBP1112 for mGluR3); and Evans Blue. A stock vehicle was prepared as a 0.1% (w/v) Evans Blue solution in 0.85% physiological saline. For administration, all active compounds were diluted in this vehicle at a ratio of 1:250, while control treatments received the vehicle without any active compounds.

### Animal allocation and injection procedure

Prior to ICV administration, chicks were weighed and randomly distributed into experimental cohorts to ensure homogeneity in initial mean body weight across all treatment groups. A single ICV injection was performed per bird using a Hamilton microsyringe without anesthetic, adhering to well-established avian protocols (Davis et al., 1979; Furuse et al., 1997). During the procedure, each bird was manually restrained with its head secured in a custom acrylic device, maintaining the skull in a 45° orientation with the calvaria parallel to the working surface (van Tienhoven and Juhasz, 1962). The implantation trajectory was directed by a pre-fabricated guide hole over the right lateral ventricle, terminating at a predetermined depth of 4 mm subjacent to the skull surface (Jonaidi and Noori, 2012). This approach is characterized by a low propensity for inducing stress-related physiological alterations in chickens (Saito et al., 2005). All compounds, including antagonists and the vehicle control, were standardized to a 10 µL volume.

### Post-injection monitoring and data acquisition

Immediately following infusion, the avian subjects were transferred back to their individual housing units, where feed and water were provided ad libitum from pre-weighed containers. Cumulative feed intake (g) was quantitatively assessed at 30, 60, and 120-minute post-infusion intervals. To mitigate the confounding influence of body mass, consumption data were normalized and are expressed relative to each subject's body weight (% BW).

### Verification of injection site and data inclusion criteria

At the conclusion of the study, euthanasia was performed on all birds. The procedure involved decapitation following the induction of deep anesthesia via an intraperitoneal administration of sodium pentobarbital (Chen et al., 2016). To confirm the precision of the ICV technique, brains were harvested and immediately frozen. Subsequent sectioning allowed for visual verification of Evans Blue dye presence within the lateral ventricle. In accordance with pre-defined criteria, data were exclusively included in the final statistical analysis for individuals confirming successful ventricular dye distribution.

### Experimental feeding schedule

This study comprised a series of 11 distinct experiments, each designed to investigate the central effects of GIP and its interference with various neural pathways on meal consumption in 3-hour-feed-deprived (FD3) broilers. A completely randomized design was employed in each experiment, involving four treatments with 12 chicks per group, resulting in a total sample size of 48 birds per experiment. The specific treatments for all experiments detailed in Table 1.

**Table 1:** Treatments procedure in experiments 1–11.

Experiment 1	ICV injection	Experiment 2	ICV injection
Treatment groups		Treatment groups	
A	Control Solution	A	Control Solution
B	GIP (3 nmol)	B	SHU9119 (MC3/MC4 receptor antagonist, 0.5 nmol)
C	GIP (6 nmol)	C	GIP (12 nmol)
D	GIP (12 nmol)	D	SHU9119+GIP
**Experiment 3**	**ICV injection**	**Experiment 4**	**ICV injection**
Treatment groups		Treatment groups	
A	Control Solution	A	Control Solution
B	HS024 (MC4 receptor antagonist, 0.5 nmol)	B	MK-801 (NMDA receptor antagonist, 15 nmol)
C	GIP (12 nmol)	C	GIP (12 nmol)
D	HS024 +GIP	D	MK-801+GIP
**Experiment 5**	**ICV injection**	**Experiment 6**	**ICV injection**
Treatment groups		Treatment groups	
A	Control Solution	A	Control Solution
B	CNQX (AMPA receptor antagonist, 390 nmol)	B	AIDA (mGLUR1 receptor antagonist, 2 nmol)
C	GIP (12 nmol)	C	GIP (12 nmol)
D	CNQX+GIP	D	AIDA+GIP
**Experiment 7**	**ICV injection**	**Experiment 8**	**ICV injection**
Treatment groups		Treatment groups	
A	Control Solution	A	Control Solution
B	LY341495 (mGLUR2 receptor antagonist, 150 nmol)	B	UBP1112 (mGLUR3 receptor antagonist, 2 nmol)
C	GIP (12 nmol)	C	GIP (12 nmol)
D	LY341495+GIP	D	UBP1112+GIP
**Experiment 9**	**ICV injection**	**Experiment 10**	**ICV injection**
Treatment groups		Treatment groups	
A	Control Solution	A	Control Solution
B	BMS193885 (NPY1 receptor antagonist, 1.25 µg)	B	CYM9484 (NPY2 receptor antagonist, 1.25 µg)
C	GIP (12 nmol)	C	GIP (12 nmol)
D	BMS193885+GIP	D	CYM9484+GIP
**Experiment 11**	**ICV injection**		
Treatment groups			
A	Control Solution		
B	L-152804 (NPY5 receptor antagonist, 1.25 µg)		
C	GIP (12 nmol)		
D	L-152804+GIP		

Control Solution: Physiological saline+ Evans Blue.

GIP: Gastric Inhibitory Polypeptide.

Experiment 1 established a dose-response relationship for GIP by evaluating the effects of ICV-administered GIP at 3, 6, and 12 nmol against a control solution. The most effective dose from this experiment was subsequently selected for all combination treatments in the following experiments.

Experiments 2 through 11 employed a consistent design to probe GIP's mechanism of action. Each of these experiments included the following four treatment arms:

• Control solution (vehicle)
• A specific receptor antagonist alone (sub-effective dose)
• GIP at the effective dose (established in Experiment 1)
• A co-administration of GIP and the antagonist

Each experiment employed the following receptor antagonists at the specified dosages:

• Experiment 2: SHU9119 (MC3/MC4 receptor antagonist, 0.5 nmol)
• Experiment 3: HS024 (MC4 receptor antagonist, 0.5 nmol)
• Experiment 4: MK-801 (NMDA receptor antagonist, 15 nmol)
• Experiment 5: CNQX (AMPA receptor antagonist, 390 nmol)
• Experiment 6: AIDA (mGluR1 receptor antagonist, 2 nmol)
• Experiment 7: LY341495 (mGluR2 receptor antagonist, 150 nmol)
• Experiment 8: UBP1112 (mGluR3 receptor antagonist, 2 nmol)
• Experiment 9: BMS193885 (NPY1 receptor antagonist, 1.25 µg)
• Experiment 10: CYM9484 (NPY2 receptor antagonist, 1.25 µg)
• Experiment 11: l-152804 (NPY5 receptor antagonist, 1.25 µg)

The selection of all pharmacological dosages was grounded in previously published empirical work (Eslam-Aghdam et al., 2024; Levine et al., 2005; Rajaei et al., 2022) and confirmed through internal, unpublished pilot studies.

### Statistical analysis

Results are presented as mean ± SEM. The primary outcome variable, cumulative feed consumption was subjected to a two-way repeated-measures analysis of variance (ANOVA). All statistical computations were performed using SPSS Statistics (Version 21, IBM Corp., Armonk, NY). In instances where the ANOVA revealed a significant main effect, post-hoc pairwise comparisons were carried out using the Tukey-Kramer procedure. Differences between treatment means were considered statistically significant at *P* < 0.05.

## Results

**Fig. 1:**
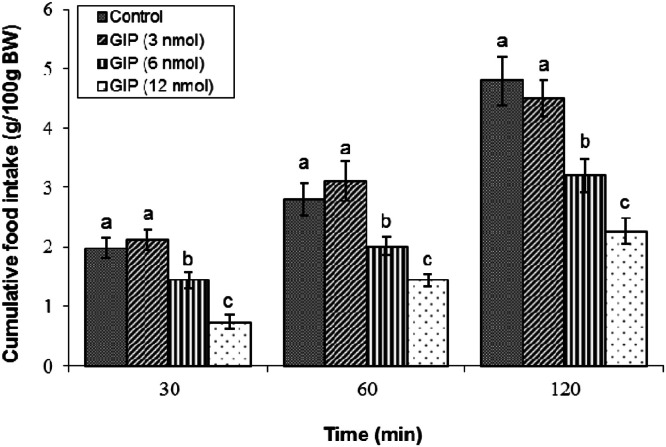
Effects of central GIP administration on cumulative feed intake in broilers. Treatment groups received: GIP (3, 6, 12 nmol) or control solution (*n* = 12 per group). Data are presented as mean ± SEM. Bars with different lowercase letters (a, b, c) differ significantly from each other (*P* < 0.05).

The anorexigenic potency of centrally administered GIP was found to be dose-dependent. ICV infusion of 3 nmol GIP failed to elicit a significant reduction in cumulative meal consumption (*P* ≥ 0.05). In contrast, both the 6 and 12 nmol doses induced significant hypophagia in 3-hour-feed-deprived broilers (*P* < 0.05). These findings were validated by ANOVA, which indicated significant main effects of treatment (F(3, 42) = 2117.32, *P* < 0.05) and time (F(2, 84) = 3159.06, *P* < 0.05), and a significant interaction (F(6, 84) = 27.37, *P* < 0.05). This led to the selection of the 12 nmol dose for all subsequent mechanistic investigations (Fig. 1).

**Fig. 2:**
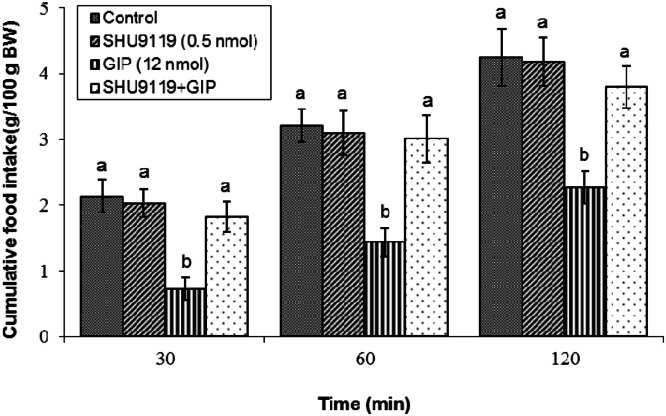
Effects of the melanocortin 3/4 receptor antagonist SHU9119 on GIP-induced hypophagia in broilers. Treatment groups received: control solution, SHU9119 (0.5 nmol), GIP (12 nmol), or their mixture (*n* = 12 per group). Data are presented as mean ± SEM. Bars with different lowercase letters (a, and b) differ significantly from each other (*P* < 0.05).

**Fig. 3:**
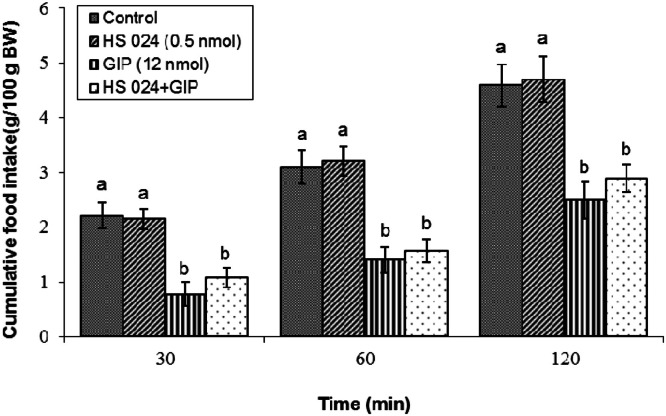
Effects of the melanocortin 4 receptor antagonist HS024 on GIP-induced hypophagia in broilers. Treatment groups received: control solution, HS024 (0.5 nmol), GIP (12 nmol), or their mixture (*n* = 12 per group). Data are presented as mean ± SEM. Bars with different lowercase letters (a, and b) differ significantly from each other (*P* < 0.05).

Pharmacological blockade of the MC3/MC4 receptors significantly attenuated GIP-induced hypophagia. Administration of a sub-effective dose of 0.5 nmol SHU9119 alone was without effect (*P* ≥ 0.05). However, its co-infusion with 12 nmol GIP significantly antagonized the peptide's hypophagic effect (*P* < 0.05). ANOVA confirmed robust effects, with significant main factors for treatment (F(3, 39) = 1781.61, *P* < 0.05) and time (F(2, 78) = 31284.09, *P* < 0.05), and a significant interaction (F(6, 78) = 29.73, *P* < 0.05) (Fig. 2). Conversely, selective inhibition of the MC4 receptor with a sub-effective dose of 0.5 nmol HS024 did not, by itself, alter feeding behavior (*P* ≥ 0.05), nor did it modify the anorexigenic response to co-administered GIP (*P* ≥ 0.05). Statistical analysis validated these results, showing significant main effects of treatment (F(3, 44) = 2759.41, *P* < 0.05) and time (F(2, 88) = 3593.71, *P* < 0.05), and a significant interaction (F(6, 88) = 26.37, *P* < 0.05) (Fig. 3).

**Fig. 4:**
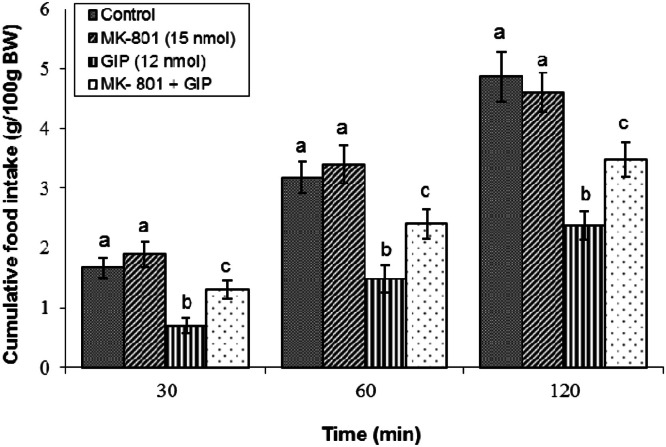
Effects of the NMDA receptor antagonist MK-801 on GIP-induced hypophagia in broilers. Treatment groups received: control solution, MK-801 (15 nmol), GIP (12 nmol), or their mixture (*n* = 12 per group). Data are presented as mean ± SEM. Bars with different lowercase letters (a, b, c) differ significantly from each other (*P* < 0.05).

Antagonism of the ionotropic NMDA receptor completely abolished the feed-suppressive effect of GIP. A sub-effective dose of 15 nmol MK-801 was ineffective alone (*P* ≥ 0.05) but partially abolished the hypophagia induced by 12 nmol GIP (*P* < 0.05). This finding was supported by ANOVA, which indicated significant main effects of treatment (F(3, 42) = 3471.38, *P* < 0.05) and time (F(2, 84) = 2753.41, *P* < 0.05), and a significant interaction (F(6, 84) = 31.59, *P* < 0.05) (Fig. 4).

**Fig. 5:**
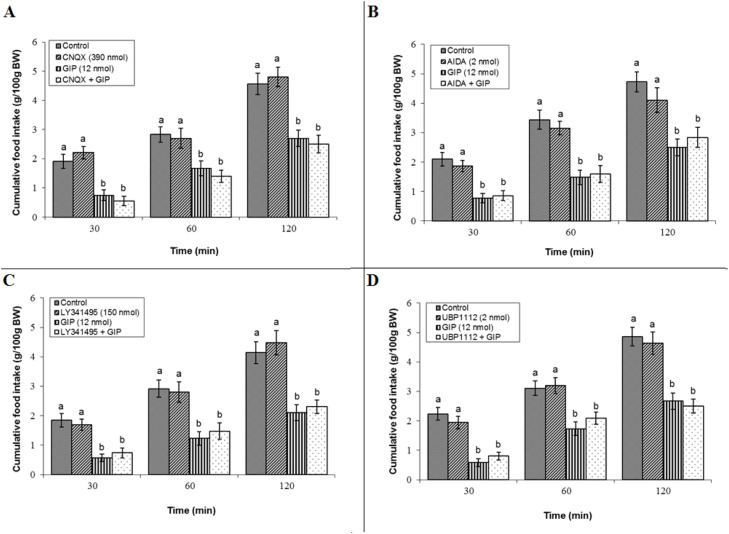
Effects of non-NMDA glutamate receptor antagonists on GIP-induced hypophagia in broilers. Treatment groups for each panel received: control, antagonist, GIP (12 nmol), or antagonist + GIP (*n* = 12 per group). Data are presented as mean ± SEM. (A) Co-infusion with the AMPA receptor antagonist CNQX (390 nmol). (B) Co-infusion with the mGluR1 receptor antagonist AIDA (2 nmol). (C) Co-infusion with the mGluR2 receptor antagonist LY341495 (150 nmol). (D) Co-infusion with the mGluR3 receptor antagonist UBP1112 (2 nmol). Bars with different lowercase letters (a, and b) differ significantly from each other within each panel (*P* < 0.05).

In contrast, a comprehensive assessment of other ionotropic and metabotropic glutamate receptors revealed no role in mediating GIP's effect. Blockade of the AMPA receptor with a sub-effective dose of 390 nmol CNQX had no intrinsic effect on consumption (*P* ≥ 0.05) and failed to attenuate GIP-induced hypophagia (*P* ≥ 0.05). The ANOVA for this experiment confirmed significant main effects of treatment (F(3, 44) = 2158.04, *P* < 0.05) and time (F(2, 88) = 3127.38, *P* < 0.05), and a significant interaction (F(6, 88) = 26.41, *P* < 0.05) (Fig. 5A). Similarly, administration of sub-effective doses of 2 nmol AIDA (mGluR1) alone yielded no significant effects (*P* ≥ 0.05), nor did it alter GIP's action (*P* ≥ 0.05). The analysis showed significant main effects of treatment (F(3, 41) = 3417.58, *P* < 0.05) and time (F(2, 82) = 3715.02, *P* < 0.05), and a significant interaction (F(6, 82) = 31.17, *P* < 0.05) (Fig. 5B). Furthermore, LY341495 (mGluR2 antagonist, 150 nmol) alone showed no effect (*P* ≥ 0.05) and failed to modify GIP's action (*P* ≥ 0.05). The two-way repeated-measures ANOVA revealed significant main effects of treatment (F(3, 44) = 3261.47, *P* < 0.05) and time (F(2, 88) = 2619.35, *P* < 0.05), and a significant interaction (F(6, 88) = 28.21, *P* < 0.05) (Fig. 5C). Finally, UBP1112 (mGluR3 antagonist, 2 nmol) did not influence feeding behavior by itself (*P* ≥ 0.05) or in combination with GIP (*P* ≥ 0.05). Analysis indicated significant main effects of treatment (F(3, 43) = 3541.24, *P* < 0.05) and time (F(2, 86) = 3018.02, *P* < 0.05), and a significant interaction (F(6, 86) = 29.05, *P* < 0.05) (Fig. 5D).

**Fig. 6:**
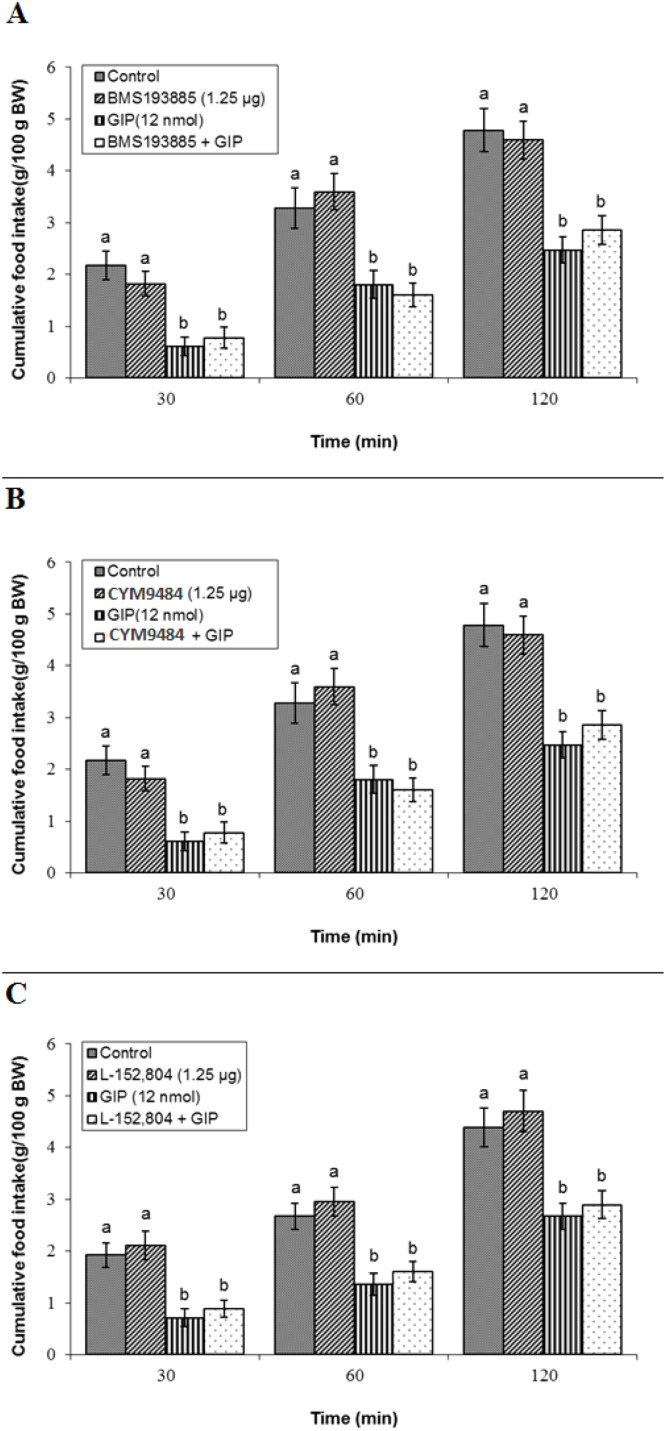
Effects of neuropeptide Y receptor antagonists on GIP-induced hypophagia in broilers. Treatment groups for each panel received: control, antagonist, GIP (12 nmol), or antagonist + GIP (*n* = 12 per group). Data are presented as mean ± SEM. (A) Co-infusion with the NPY Y1 receptor antagonist BMS193885 (1.25 µg). (B) Co-infusion with the NPY Y2 receptor antagonist CYM9484 (1.25 µg). (C) Co-infusion with the NPY Y5 receptor antagonist l-152,804 (1.25 µg). Bars with different lowercase letters (a, and b) differ significantly from each other within each panel (*P* < 0.05).

Central NPY receptor signaling was not found to mediate GIP's anorexigenic action. Independent infusion of a sub-effective dose of BMS193885 (NPY1 receptor antagonist, 1.25 µg) did not affect meal consumption alone (*P* ≥ 0.05), and its co-infusion with GIP left the peptide's hypophagic effect intact (*P* ≥ 0.05). ANOVA confirmed significant main effects of treatment (F(3, 44) = 2541.12, *P* < 0.05) and time (F(2, 88) = 1786.18, *P* < 0.05), and a significant interaction (F(6, 88) = 22.14, *P* < 0.05) (Fig. 6A). Likewise, CYM9484 (NPY2 receptor antagonist, 1.25 µg) alone was without effect (*P* ≥ 0.05) and did not attenuate GIP-induced hypophagia (*P* ≥ 0.05). Statistical analysis revealed significant main effects of treatment (F(3, 42) = 2813.40, *P* < 0.05) and time (F(2, 84) = 3159.31, *P* < 0.05), and a significant interaction (F(6, 84) = 24.41, *P* < 0.05) (Fig. 6B). Finally, l-152,804 (NPY5 receptor antagonist, 1.25 µg) did not alter basal feeding (*P* ≥ 0.05) nor modify the response to GIP (*P* ≥ 0.05). The ANOVA indicated significant main effects of treatment (F(3, 43) = 2571.38, *P* < 0.05) and time (F(2, 86) = 3062.41, *P* < 0.05), and a significant interaction (F(6, 86) = 27.15, *P* < 0.05) (Fig. 6C).

## Discussion

The present investigation provides novel and mechanistic insights into the central pathways through which GIP modulates food intake in neonatal broilers. This work moves beyond phenomenological description by employing a systematic approach to identify, for the first time in an avian model, a specific "GIP-melanocortin-NMDA receptor" axis as a critical neurocircuit for the anorexigenic action of this gut-derived hormone. Our findings robustly demonstrate that the hypophagic effect of centrally administered GIP is dose-dependent and necessitates functional signaling through both melanocortin (MC3/4) and NMDA glutamate receptors, while definitively excluding a necessary role for MC4, AMPA, metabotropic glutamate, and NPY Y1/Y2/Y5 receptors in this acute response. This delineation of a defined neurochemical pathway for GIP in the avian brain represents a significant advance in our understanding of comparative neuroendocrinology.

The observed anorexigenic effect of GIP is congruent with an emerging body of evidence from mammalian studies that firmly establishes central GIPR signaling as a physiologically relevant regulator of energy homeostasis (Zhang et al., 2021; Lewis et al., 2024). The dose-dependent relationship we observed, with significant hypophagia at 6 and 12 nmol but not at 3 nmol, is particularly insightful. It mirrors findings in mice, where a threshold dose of GIP was required to suppress feeding (NamKoong et al., 2017), suggesting a conserved pharmacodynamic profile across vertebrate classes. This specificity argues against a non-specific or toxic effect and strengthens the case for a receptor-mediated mechanism. Collectively, our data extend the physiological relevance of central GIP signaling from mammals to birds, suggesting a deeply conserved role for this peptide in vertebrate energy balance that had previously been characterized primarily in mammalian models.

A principal finding of this investigation is the identification of the central melanocortin system as a critical mediator of GIP-induced hypophagia. The potent attenuation of GIP's anorexigenic effect by the dual MC3/4R antagonist SHU9119 provides direct pharmacological evidence for this interaction. This finding is strongly supported by a compelling body of evidence from mammalian models. GIPR is robustly expressed in pivotal hypothalamic regions governing energy intake, including the ARC (Adriaenssens et al., 2019), and its activation is necessary for GIP's central anorectic effects (Zhang et al., 2021). Crucially, GIPR activation has been shown to stimulate POMC neurons, the primary source of anorexigenic α-MSH (Yada, 2024; Zhang et al., 2021). Furthermore, a recent seminal study by Han et al. (2023) provided direct mechanistic evidence by demonstrating that a long-acting GIPR agonist (GIPFA-085) increases cytosolic Ca²⁺ in ARC leptin-responsive and POMC neurons, and that GIP and leptin cooperate to activate these neurons and inhibit food intake. The critical downstream role of MC4R is well-documented across species. MC4R is a principal regulator of satiety, as evidenced by the hyperphagia and obesity in MC4R-deficient models (Huszar et al., 1997) and the orexigenic effects of its antagonists (Kask et al., 1999). In contrast, MC3R deletion does not alter meal consumption (Chen et al., 2000). Therefore, our data position GIP as a novel upstream activator of this conserved ARC POMC → MC4R satiety pathway in avian species, aligning with the recognized paradigm of central melanocortin-mediated appetite regulation (Bungo et al., 2011; Honda et al., 2012).

A second major finding that constitutes a key scientific advance of this work is the identification of NMDA receptor-mediated glutamatergic transmission as an indispensable downstream effector within the central GIP signaling cascade. The complete abolition of GIP-induced hypophagia by the NMDA receptor antagonist MK-801 demonstrates that NMDA receptor activation is not merely permissive but is a necessary component of GIP's anorexigenic action, a finding consistent with the established role of NMDA receptors in mediating satiety signals in other contexts (Campos and Ritter, 2015; Duva et al., 2005). This discovery allows us to propose a novel, integrated neurocircuitry for GIP. We hypothesize that the anorexigenic signal initiated by GIP is either carried by intrinsically glutamatergic GIPR neurons or potently modulates glutamatergic synapses within a dedicated satiety circuit, ultimately requiring NMDA receptor activation to suppress feeding. This model is powerfully supported by molecular evidence showing that the majority of GIPR-expressing neurons in the mouse hypothalamus are glutamatergic (Adriaenssens et al., 2019) and that glutamate itself acts as a primary anorexigenic neurotransmitter in the avian brain (Baghbanzadeh and Babapour, 2007; Ahmadi et al., 2019). The potential for crosstalk and synergy within this circuit is a compelling prospect. For instance, leptin, another potent anorexigenic hormone, has been shown to potentiate NMDA receptor function (Harvey et al., 2005) and to enhance NMDA receptor-dependent synaptic plasticity (Lau and Zukin, 2007), suggesting that the NMDA receptor may serve as a key integrative node where multiple satiety signals, including GIP and leptin, converge to synergistically amplify glutamatergic tone and suppress feeding. This convergence is particularly plausible given that both GIP (present study) and leptin (Srour et al., 2024) engage POMC neurons in the ARC, which are known to be regulated by glutamatergic inputs. Therefore, our data definitively position the NMDA receptor not as a parallel pathway, but as an integral component of a defined "GIP-melanocortin-glutamate" axis, resolving a key mechanistic question about how this gut-derived signal engages central excitatory neurotransmission to control appetite.

In stark contrast to the clear contributions of the melanocortin and glutamatergic systems, our comprehensive pharmacological approach definitively excluded a necessary role for the primary orexigenic NPY receptor subtypes (Y1, Y2, and Y5) in mediating the acute hypophagic effect of GIP. The failure of selective antagonists for these receptors to attenuate GIP's action is a critical and highly informative finding. Given the potent and well-characterized orexigenic drive mediated by Y1 and Y5 receptors in the avian brain (Mahdavi et al., 2024; Yousefvand et al., 2019; Greene et al., 2022), this result demonstrates that the rapid anorectic signal generated by GIP operates through a dedicated satiety circuit that is functionally segregated from the primary NPY-mediated hunger pathway. This functional segregation is a key physiological insight, suggesting that GIP does not act as a simple brake on ongoing hunger signals but instead engages a selective, hard-wired anorexigenic program, a principle consistent with findings in mammals where distinct neural populations regulate feeding in a non-redundant manner (Li et al., 2019). This specificity is crucial as it allows for the independent regulation of satiety and hunger, a feature that could be leveraged for more targeted metabolic interventions with fewer side effects. The apparent independence of GIP from NPY signaling in broilers also highlights potential species-specific differences in gut-brain axis communication, as a study in dogs reported that central NPY infusion could stimulate GIP secretion (Yavropoulou et al., 2008), suggesting a bidirectional relationship not observed in our acute model. Collectively, this clear dissociation between the GIP-driven satiety pathway and the core NPY orexigenic system delineates distinct neurochemical architectures for hunger and satiety in the avian brain, refining our comprehension of how specific gastrointestinal signals are centrally processed to modulate energy homeostasis.

## Conclusion

In conclusion, this study fundamentally advances the field of avian appetite regulation by delineating a novel and evolutionarily conserved neurocircuitry for central GIP. We have established that its hypophagic effect is non-redundant, requiring sequential signaling through melanocortin receptors and downstream NMDA-type glutamate receptors, while being independent of NPY Y1, Y2, and Y5 receptor signaling. The identification and characterization of this "GIP-melanocortin-NMDA receptor" axis is the key scientific advance of this work. It provides a concrete and testable mechanistic framework that resolves the previously unknown central action of GIP in birds and offers fresh insights into the integration of gut-derived signals with central feeding circuits. Future research should aim to map the precise neuroanatomical connectivity of this circuit, investigate its modulation by physiological energy status, and explore its interactions with other gut-brain axes. From an applied perspective, this axis represents a potential target for strategies aimed at modulating feed efficiency and growth in poultry production.

## Consent for publication

Not applicable.

## Availability of data and materials

The data used and/or analyzed during the current study is available from the corresponding author on reasonable request.

## Funding

This research did not receive any specific grant from funding agencies in the public, commercial, or not-for-profit sectors.

## Ethics approval and consent to participate

This study was approved by the School of Veterinary Medicine at the University of Tehran, Iran (ethical code: IR.UT.VETMED.REC.1403.061) and according to Iranian government and the National Institutes of Health's Guide for the Care and Use of Laboratory Animals (publication No. 85-23, revised in 1996).

## Declaration of generative AI and AI-assisted technologies in the writing process

During the preparation of this work the author(s) used Perplexity AI in order to identify and correct potential grammatical errors and improve the overall flow and readability of the manuscript. After using this tool, the author(s) reviewed and edited the content as needed and take(s) full responsibility for the content of the published article.

## CRediT authorship contribution statement

**Maryam Lotfi Gharaie:** Writing – original draft, Methodology, Investigation. **Morteza Zendehdel:** Writing – review & editing, Supervision, Formal analysis, Conceptualization. **Hamed Zarei:** Conceptualization, Methodology, Writing – review & editing. **Kimia Mahdavi:** Writing – review & editing, Conceptualization.

## Disclosures

The authors report no conflicts of interest.
